# Application of Low Temperature during the Malaxation Phase of Virgin Olive Oil Mechanical Extraction Processes of Three Different Italian Cultivars

**DOI:** 10.3390/foods10071578

**Published:** 2021-07-07

**Authors:** Gianluca Veneziani, Davide Nucciarelli, Agnese Taticchi, Sonia Esposto, Roberto Selvaggini, Roberto Tomasone, Mauro Pagano, Maurizio Servili

**Affiliations:** 1 Department of Agricultural, Food and Environmental Sciences, University of Perugia, Via S. Costanzo, 06126 Perugia, Italy; gianluca.veneziani@unipg.it (G.V.); davide.nucciarelli@unipg.it (D.N.); agnese.taticchi@unipg.it (A.T.); roberto.selvaggini@unipg.it (R.S.); maurizio.servili@unipg.it (M.S.); 2 Council for Agricultural Research and Economics Research, Centre for Engineering and Agro-Food Processing, 00015 Monterotondo, Italy; roberto.tomasone@crea.gov.it (R.T.); mauro.pagano@crea.gov.it (M.P.)

**Keywords:** low temperature, malaxation, VOO quality, hydrophilic phenols, volatile compounds

## Abstract

The malaxation step, one of the most important phases of the virgin olive oil (VOO) mechanical extraction process involved in the development of the main quality characteristics of the final product, was carried out at a low temperature (18 °C). The rapid control of malaxer temperature was handled with the same chiller as that of the heat exchanger used in a semi-industrial extraction plant. Low temperature was used during the full olive paste kneading process and also for half of this process, which showed that there was a significant impact on the phenolic and volatile contents of VOO. Trials were conducted on three different cultivars (Canino, Moraiolo and Peranzana), and their phenolic and volatile concentrations showed different quantitative and qualitative effects due to the prolonged use of low temperature after the crushing phase, as a function of the different genetic origins of the olives. The process of phenolic compound solubilization into the oily phase was negatively influenced by the use of low temperature during the entire malaxation period for all the cultivars, whereas the volatile fraction showed an improvement in VOO flavor mainly due to the oil extracted from Canino olives.

## 1. Introduction

In recent years, the thermal conditioning of olive paste during the olive oil extraction process has increased in usage due to the different impact in oil yield and the quality characteristics of the final product. Previous studies focused their attention on the biochemical process, showing that a differentiated activity level of endogenous enzymes occurred due to the application of different processing temperatures [1,2,3,4,5,6]. The possibility of accurate thermal control and management of temperature conditions during crushing and malaxation phases stimulated the investigation of different cultivar behaviors during the virgin olive oil (VOO) extraction process [7,8], to discover the best combinations of process parameters that influence the chemical characteristics and sensorial properties affecting VOO quality. The optimization of technological variables plays an important role in the modulation of physical, chemical and biochemical processes with the aim of improving VOO qualitative standards. One of the most important parameters of the VOO mechanical extraction process is the processing temperature. This parameter can modify and regulate the different enzyme activities due to the endogenous enzymes present in the constitutive parts of olive drupe, such as depolymerizing enzymes (pectinase, cellulase and hemi-cellulase), poly-phenol-oxydase (PPO), peroxidase (POD) and lipoxygenase (LOX). The possibility of controlling the temperature during the entire extraction process allows the opportunity to regulate the biochemical reactions of some endogenous enzymes that have a high impact on VOO quality, which is related to the health and sensory characteristics of the final product [4,5].

The application of high temperature favors the degradative process of phenolic compounds induced by PPO and POD and promotes enzymatic activities, which increases the oxidation process during the mechanical extraction of VOO. The high temperature of fruits before the extraction process, the increasing temperature of olive paste during crushing and, most of all, the regulation of malaxation temperature are all factors that can strictly influence the activity level of PPO and POD. For this reason, many studies have been performed to optimize the processing temperature in order to partially control the final phenolic content of the product during the different steps of the extraction process [3,4,9].

On the other hand, the most important fraction of volatile compounds that guarantees the development of VOO flavor is obtained in the first phases of the technological process, during crushing and immediately thereafter. A minor percentage is also produced during the kneading phase of olive paste, as reported in previous studies [10,11]. The lipoxygenase pathway extracted from olive fruit showed a high activity level at temperatures ranging from 15 °C to 40 °C [12,13], but the best performances were obtained at temperatures ≤ 20 °C [13,14,15]. These results were mainly due to the optimum temperature of hydroperoxide lyase (HPL), which was detected between 15 °C and 20 °C and showed a high increase in C_5_ and C_6_ compounds [13]. As demonstrated by different studies, employing the thermal treatment of olives and/or crushed olive pastes with a low temperature increased the concentration of volatile compounds, improving the fruity and herbaceous sensory notes of VOO extracted from different olive cultivars [14,16,17]. However, the impact was highly differentiated, strictly related to the genetic origin of the fruits and influenced by the specific lipoxygenase activity load of the cultivar [8,9,10,11]. The quantitative and qualitative variability of the volatile fractions of saturated and unsaturated C_5_ and C_6_ aldehydes, alcohols and esters was influenced not only by the cultivars but also by other factors, such as the ripening stages of the fruit, agricultural practices, growing areas, and climatic and technological aspects [18,19].

Currently, the best way to manage the temperature of extraction plants at an industrial scale is to employ the rapid conditioning of olive paste after the crushing step with a heat exchanger [8,17,20,21,22]. The main industrial manufacturers of VOO extraction systems produce different models of heat exchanger, which are fully used by operators in the VOO sector.

Probably due to the different temperature values, this group of endogenous enzymes works differently, and an interesting research topic is to examine how the specific activity that characterizes each different cultivar is influenced by the olive oil paste exposure time at low temperature. The research design was carried out on three different cultivars characterized by different concentrations of phenolic and volatile compounds, from a quantitative and qualitative point of view, in order to better evaluate the impact of innovative technology on VOOs obtained by olive fruits of different genetic origin. The main aim of the work concerned the use of temperatures below 20 °C during the malaxation phase to assess the potential effect on the development of volatile compounds and on the phenol content of VOO in relation to the different genetic origins of the raw material.

## 2. Materials and Methods

### 2.1. Chemicals

The seco-iridoid derivatives and lignans were extracted from VOO, as described by Selvaggini et al. (2014). The chemical standards, solvents and other compounds used during the analyses of VOO were supplied by Merck (Merck KGaA, Darmstadt, Germany), tyrosol was supplied by Cabru s.a.s. (Arcore, Milan, Italy) and hydroxy-tyrosol was supplied by Fluka (Milan, Italy).

### 2.2. VOO Mechanical Extraction Process

The olives belonged to three different Italian cultivars (Canino, Moraiolo and Peranzana) and were processed within 48 h after the harvesting period and from the middle of October to the first week of November 2019. The olive growing areas of Canino and Peranzana were located in the Lazio and Apulia regions, respectively, whereas the olives of Moraiolo were harvested in the Umbria region. VOOs were extracted from approximately 180 kg of olives using an industrial plant TEM 200 system (Toscana Enologica Mori, Tavarnelle Val di Pesa, Florence, Italy) equipped with a hammer mill, a malaxer with a gas controller system that had a working capacity of 200 kg of olives, and a two-phase decanter. The final separation step was carried out with a UVPX 305 AGT 14 centrifuge (Alfa Laval S.p.A., Tavarnelle Val di Pesa, Florence, Italy). Rapid control of olive paste after the crushing phase was obtained using an EVO-Line heat exchanger (Alfa Laval S.p.A.). The same chiller system as the heat exchanger was also used to regulate the temperature of the malaxer during the 30 min kneading phase. The control trials (C-VOO) were conducted at 25 °C, whereas the experimental tests were conducted by settling the temperature of the heat exchanger at 18 °C and changing the temperature of the malaxer as follows:

- Control (C-VOO) heat exchanger at 25 °C, malaxer at 25 °C for 30 min;
- Test 1 (T1-VOO) heat exchanger at 18 °C, malaxer at 25 °C for 30 min;
- Test 2 (T2-VOO) heat exchanger at 18 °C, malaxer at 18 °C for 30 min;
- Test 3 (T3-VOO) heat exchanger at 18 °C, malaxer at 18 °C for 15 min + 15 min at 25 °C.

### 2.3. VOO Chemical Analysis

#### 2.3.1. Legal Quality Parameters

The main legal quality parameter methods (free fatty acids, peroxide value, K232, K270 and ΔK) were evaluated according to Regulation (EU) 2015/1830 [23].

#### 2.3.2. Phenolic Compounds

The content of the main hydrophilic phenols of VOO controls and extracts at low temperature was analyzed with high-performance liquid chromatography (HPLC) using the same instrument and the same method as described by Taticchi et al. (2021). The concentrations of 3,4-DHPEA-EDA (oleacein), p-HPEA-EDA (oleocanthal), 3,4-DHPEA-EA (isomer of the oleuropein aglycon), p-HPEA-EA (ligstroside aglycon), 3,4-DHPEA (hydroxy-tyrosol), p-HPEA (tyrosol), vanillic acid, p-cumaric acid, (+)-1-acetoxypinoresinol and (+)-pino-resinol were expressed in mg/kg of oil.

#### 2.3.3. Volatile Compounds

The volatile fractions of the VOO control and extracts at low temperature were analyzed with headspace solid-phase microextraction followed by gas chromatography–mass spectrometry (HS-SPME/GC-MS) using an Agilent Technologies GC 7890B instrument equipped with a “Multimode Injector” (MMI) 7693A (Agilent Technologies, Santa Clara, CA, USA) and followed the method described by Taticchi et al. [24]. The concentrations of aldehydes (pentanal, (E)-2-pentenal, hexanal, (E)-2-hexenal, (E,E)-2,4-hexadienal), alcohols (1-pentanol, 1-penten-3-ol, (E)-2-penten-1-ol, (Z)-2-penten-1-ol, 1-hexanol, (E)-2-hexen-1-ol, (Z)-3-hexen-1- ol), esters (hexyl acetate (Z)-3-hexenyl acetate) and ketones (3-pentanone, 1-penten-3-one and 6-methyl-5-hepten-2-one) were expressed in µg/kg of oil.

### 2.4. Statistical Analysis

All the phenolic and volatile compound data of the VOO controls and experiments were analyzed by SigmaPlot Software 12.3 (Systat Software Inc., San Jose, CA, USA) with one-way analysis of variance (ANOVA).

## 3. Results and Discussion

Based on the results, in the mechanical extraction process of VOO, the use of low temperature during the first phases, such as the crushing and malaxation of olive paste phases, showed that the temperature had a significant role in controlling the activities of the main endogenous enzymes (LOX, PPO and POD) that regulate both the neoformation of volatile compounds and the oxidation phenomenon of the phenolic fraction [3,5,8,16,17]. For these reasons, the research design compared three different cultivars to better assess the effects of the low temperature in relation to different olive fruits characterized by different levels of enzymatic activity and different concentrations of phenolic and volatile compounds. Currently, the use of a heat exchanger to decrease the temperature after the crushing step is widespread, and its chiller system has also been utilized to reduce the temperature of the malaxer, which extended the treatment at low temperature during the kneading of olive paste in order to evaluate its impact on VOO quality.

The use of low temperature during the malaxation phase did not modify the oil yield, which confirmed the data reported in a previous study that did not observe obstacles to the coalescence phenomena of oil droplets and VOO extractability [17].

Compared with the control test results, the assessment of the quality indices of T1, T2 and T3-VOOs did not show significant differences for all three cultivars, with values of free fatty acids, peroxide, K_232_, K_270_ and ΔK in line with the legal limit [23] (Table 1).

### 3.1. Test 1―Use of Low Temperature after Crushing Phase

The use of flash thermal conditioning of olive paste after the crushing phase (T1-VOO), induced by a heat exchanger, allowed for the positive variation in the phenolic fraction to be determined, which was a function of the genetic origin of the olives. The settling of the heat exchanger at 18 °C confirmed data that indicated a significant improvement in the phenolic composition for the T1-VOOs extracted from the three cultivars in comparison with the C-VOOs [8]. The phenolic content recorded increases of 44.3%, 82% and 14.8% for the Canino, Moraiolo and Peranzana cultivars, respectively (Table 2).

The high enhancement of Canino was very interesting because the control content of phenolic compounds was very low and the use of a heat exchanger highly improved the sensory and health properties of the product.

The rapid reduction in the temperature of crushed olive paste (T1-VOO) also confirmed a significant influence of the cultivar on volatile compounds. The effects were strictly related to the different levels of the activity of the lipoxygenase (LOX) pathway enzymes that regulate the development of the VOO volatile fraction [11,25].

For example, the T1-VOO of Canino showed a slight increase in the sum of saturated and unsaturated C_5_ and C_6_ aldehydes when compared to C-VOO, which was a difference that was not detected for the other two cultivars (Table 3). However, a slight reduction trend was common for the sum of the saturated and unsaturated C_5_ and C_6_ alcohols of T1-VOO in all the cultivars in comparison to that of the control oil extracted at 25 °C. The ester concentration, which was very low during the 2019 harvesting season, also existed in the cultivars, and was usually characterized by high content, such as in the case of Peranzana [7,26], showing a reduction of content in Canino VOO and a slight increase in Peranzana VOO in Test 1 (Table 3, Table 4 and Table 5).

### 3.2. Tests 2 and 3―Use of Low Temperature during the Malaxation Phase

The other two experimental trials (Tests 2 and 3) also prolonged the use of low temperature during the malaxation phase to evaluate the eventual positive effects on the formation of volatile compounds and on the hydrophilic phenol content of VOOs. The data obtained from Test 2 and Test 3 showed a differentiated impact on VOO quality parameters due to the different cultivars, probably as a consequence of the different activities of endogenous enzymes that regulate volatile and phenolic compounds. In fact, the LOX pathway is characterized by different levels of enzymatic activity, which are influenced by the genetic origin of the fruits, and this explains the variable development of the volatile aldehydes, alcohols and ester compounds that determine the large aromatic variability of VOO, as explained by other authors [25,27].

The different LOX activities are influenced not only by agronomic practices, the growing area, and the ripening index but also by the temperature of the extraction process [4,8,11,28]. With the use of low temperature during the entire cycle of malaxation (T2-VOO) and by extending the time of optimum temperature of LOX activity [29], a further significant increase in (E)-2-exenal in VOO of the Canino cultivar was observed. The sum of the aldehydes mainly showed an enhancement of 48.9% and 33.9% of T2-VOO compared with the enhancements seen in C-VOO and T1-VOO (Table 3). On the contrary, alcohols showed an opposite trend, with a significant reduction in the sum of C_5_- and C_6_-saturated and unsaturated alcohols of 24.5% compared to the C-VOO.

Although significant differences have been pointed out among the different tests, the concentration of esters of Canino VOO was probably too low to recognize a variability in the sensory properties of the product [30,31]. When reducing the period of low temperature during the malaxation phase to 15 min (T3-VOO), the volatile fraction of the Canino cultivar showed a decrease in the sum of aldehydes compared with T2-VOO but a higher value compared with C-VOO and T1-VOO (Table 3). The results of the Canino cultivar suggest a close connection between the increase in aldehydes and the use of low temperature during the crushing and malaxation phases, which could have significant enhancements depending on the time spent by olive paste at 18 °C. The prolongation of low temperature seems to improve the activity of specific hydroperoxide lyases that generate the main C_6_ aldehydes. In contrast, the application of low temperature during the kneading of olive paste showed a significant impact on the volatile sensory note of Canino VOO with an increase in green and leaf-like aroma due to (E)-2-exenal [30] and a reduction in fruity and green sweet sensory notes due to the main alcohols [29,32]. Similar data and the high influence of cultivars on different synthesis of C_5_ and C_6_ volatile compounds was also recently proposed by other authors [6,33,34].

On the other hand, the use of low temperature during the malaxation phase reduced the phenolic content of Canino T2-VOO by 34% compared to T1-VOO, whereas the value rose again in Test 3 when the low temperature was maintained for only half of the kneading process. The data highlighted that the inhibitory effect of low temperature on PPO and POD activity [5] was minor compared to the effect of the solubilization process of phenolic compounds in the oily phase induced by the application of higher temperature (25 °C) during the malaxation step (Table 2). Qualitatively, the main differences were observed for oleuropein and ligstroside derivatives such as 3,4-DHPEA-EDA, p-HPEA-EDA and 3,4-DHPEA-EA. The VOO phenolic concentrations of the other two cultivars (Moraiolo and Peranzana) showed the same trend as Canino VOOs (Table 2). The prolonged use of low temperature for the entire cycle of the malaxation step (Test 2) confirmed that the reduction of partition processes in the phenolic fraction between aqueous and oily phases of olive paste occurred with decreases of 18.3% and 21.4% for T2-VOO hydrophilic phenol content compared to T1-VOOs for Moraiolo and Peranzana. However, the reduction trend was more limited if the results of T2-VOOs were compared with the C-VOOs, and there were differences that were not statistically significant for Canino and Peranzana cultivars. These results were significant in confirming the high impact of the rapid thermal conditioning of crushed olive paste (Test 1), which highlighted the importance of the use of a heat exchanger in oil mechanical extraction plants to improve the phenolic fraction of VOOs and, as a consequence, the health and sensory properties of the product [8,17,20,21,22].

Another interesting result, which was also confirmed by the Moraiolo and Peranzana cultivars, concerned the evolution of VOO phenolic compounds during Test 3, in which increasing the temperature at 25 °C for the last 15 min of malaxation time was enough again to improve the levels of solubilization of the phenolic fraction into the oily phase of olive paste. The T3-VOOs enhanced their concentration of 8% and 12.4% compared with the trials conducted at 18 °C for the entire malaxation time for Moraiolo and Peranzana cultivars (Table 2).

In contrast, the genetic origin of the fruits had a major influence on the evolution of VOO volatile compounds both in quantitative and qualitative terms. In fact, the impact of different temperatures used after the crushing phase or during malaxation highlighted different evolution trends for the other two cultivars compared with the concentrations of the volatile fraction of Canino VOOs. The sum of aldehydes in Test 2 slightly increased, and above all in the Moraiolo VOO, even if the data were not statistically significant. Concerning the sum of alcohols and esters, the results showed a reduction and an increase in their concentration, respectively, compared to the C-VOO of Peranzana. The alcohols of Moraiolo showed the same evolution trend even if the data were not statistically significant, whereas the ester content, as analyzed for Canino VOOs, was too low to recognize the effect of a significant increase (Table 4 and Table 5). During the technological tests on the optimization of the oil mechanical extraction process, the use of low temperature showed a constant impact on the VOO phenolic content, whereas the evolution of volatile compounds seemed to be more complex with different responses in function for the different olives processed. As reported by other studies on VOO technology, the quantitative and qualitative volatile fraction is first influenced by the genetic origin of the fruits, which are characterized by different behaviors when different technological parameters are modified [4,8,20,24,25,26,27,33,34,35,36,37,38].

## 4. Conclusions

The first result of this study confirmed the positive role of rapid thermal conditioning on the olive paste of different cultivars after the crushing phase, which was induced by the reduction of temperature using a heat exchanger. The impact was mainly related to the improvement of the hydrophilic phenol content of VOOs in all the cultivars processed with different quantitative increases. In contrast, the low temperature, also applied during the malaxation phase, determined an overall reduction in the phenolic fraction. The data highlighted how the solubilization phenomenon of phenolic compounds into the oily phase, promoted at 25 °C of extraction temperature, played a higher role in the determination of the final VOO phenolic concentration than the impact of a probable reduction in the enzymatic oxidation induced by the use of lower temperature during the kneading of olive paste.

The impact on the volatile fraction, which influenced the VOO flavor, was strongly conditioned by the genetic origin of the olives, which determined different responses to the application of low temperature during the malaxation phase. The VOO of the Canino cultivar was the only cultivar that showed a significant evolution of the volatile fraction, with a higher content of the sum of aldehydes and a reduction of the sum of alcohols that contributed to an enhancement of the “green” sensory note of VOOs. The use of low temperature for half of the malaxation phase could represent, for the Canino cultivar, a middle course to optimize the reduction effect of phenolic compounds and the increase of aldehydes, whereas for the other cultivars, which did not show significant improvement in volatile concentration, application of 18 °C temperature during the kneading step would not be recommended.

Knowledge of new technologies and the settling of different technological parameters, such as temperature and time of treatment, should be implemented to optimize the mechanical extraction process as a function of the different cultivars processed, with the opportunity of obtaining VOO of higher quality standards. The high variability was also apparent in this study and mainly related to the volatile content evolution. This variability suggests the importance of having a range of solutions to extract from each different cultivar, different VOOs characterized by different levels of volatile and phenolic compounds that can characterize the sensory and health properties of the product and intercept with different groups of consumers.

## Figures and Tables

**Table 1:** Legal quality parameters of VOOs extracted at different processing temperatures.

	C-VOO	T1-VOO	T2-VOO	T3-VOO
	Canino			
Free fatty acid (% oleic acid) ^1^	0.29 ± 0.01a	0.28 ± 0.01a	0.26 ± 0.01a	0.26 ± 0.01a
Peroxide value (meqO_2_/kg)	8.9 ± 2.8a	6.8 ± 1.1a	8.4 ± 0.3a	6.7 ± 0.1a
K_232_	1.78 ± 0.32a	1.63 ± 0.04a	1.71 ± 0.03a	1.64 ± 0.08a
K_270_	0.11 ± 0.004a	0.13 ± 0.01a	0.12 ± 0.01a	0.14 ± 0.003a
∆K	−0.002 ± 0.0004a	−0.002 ± 0.0002a	−0.002 ± 0.0004a	−0.003 ± 0.0004a
	Moraiolo			
Free fatty acid (% oleic acid) ^1^	0.29 ± 0.01a	0.29 ± 0.01a	0.32 ± 0.02a	0.3 ± 0.01a
Peroxide value (meqO_2_/kg)	6.1 ± 1.1a	5.5 ± 0.3a	7.3 ± 1.3a	5.8 ± 0.5a
K_232_	1.81 ± 0.08a	1.72 ± 0.04a	1.72 ± 0.1a	1.68 ± 0.02a
K_270_	0.12 ± 0.01a	0.12 ± 0.01a	0.12 ± 0.01a	0.11 ± 0a
∆K	−0.003 ± 0.0004a	−0.003 ± 0.0004a	−0.003 ± 0.0004a	−0.003 ± 0.0004a
	Peranzana			
Free fatty acid (% oleic acid) ^1^	0.28 ± 0.01a	0.28 ± 0.01a	0.3 ± 0.01a	0.3 ± 0.01a
Peroxide value (meqO_2_/kg)	8.1 ± 2.3a	8.2 ± 2.6a	8.9 ± 0.7a	6.4 ± 0.1a
K_232_	2.07 ± 0.21a	1.9 ± 0.16a	1.93 ± 0.08a	1.71 ± 0.02a
K_270_	0.16 ± 0.002a	0.14 ± 0.01a	0.15 ± 0.001a	0.12 ± 0.002a
∆K	−0.002 ± 0.0002a	−0.002 ± 0.003a	−0.003 ± 0.0002a	−0.002 ± 0.0004a

^1^ The data are the mean values of two independent extractions, ± standard deviation. The values in each row having different letters (a) are significantly different from one another (*p* < 0.05). C = control (heat exchanger at 25 °C, malaxer at 25 °C for 30 min); T1 = test 1 (heat exchanger at 18 °C, malaxer at 25 °C for 30 min); T2 = test 2 (heat exchanger at 18 °C, malaxer at 18 °C for 30 min); T3 = test 3 (heat exchanger at 18 °C, malaxer at 18 °C for 15 min + 15 min at 25 °C).

**Table 2:** Phenolic composition (mg/kg) of VOOs extracted at different processing temperatures.

	C-VOO	T1-VOO	T2-VOO	T3-VOO
	Canino			
3,4-DHPEA ^1^	1.2 ± 0.1a	1.1 ± 0.1a	1.1 ± 0.8a	1.9 ± 0.1a
*p*-HPEA	1.1 ± 0.2a	1.6 ± 0.5ab	1.5 ± 0.1ab	2.4 ± 0b
Vanilic acid	0.1 ± 0a	0.2 ± 0.1a	0.2 ± 0a	0.3 ± 0a
*p*-Cumaric acid	n.d.	n.d.	n.d.	n.d.
3,4-DHPEA-EDA	87.6 ± 5.4a	129.2 ± 14.1b	78.4 ± 3.6a	126.5 ± 5.8b
*p*-HPEA-EDA	20 ± 0.6a	31.4 ± 2.4b	19 ± 1.3a	26.8 ± 2bc
(+)-1-acetoxypinoresinol	9.8 ± 1.1a	11.1 ± 0.8a	10.1 ± 1.1a	12 ± 0.1a
(+)-pinoresinol	10.4 ± 1.1a	11.3 ± 0a	9.2 ± 1.4a	12 ± 1a
3,4-DHPEA-EA	9.4 ± 0.8a	15.2 ± 1.3b	9.9 ± 1.3a	22.6 ± 0.6c
Ligstroside aglycone	6 ± 0.3a	9 ± 3.3a	8.3 ± 2.1a	9.3 ± 0.4a
Total phenols	145.6 ± 5.8a	210.1 ± 14.8b	137.7 ± 5.1a	213.8 ± 6.3b
	Moraiolo			
3,4-DHPEA ^1^	0.9 ± 0.2a	1 ± 0.1a	1 ± 0a	1.3 ± 0.2a
*p*-HPEA	1.2 ± 0.4a	1.3 ± 0.1a	1.7 ± 0.1a	1.6 ± 0a
Vanilic acid	0.2 ± 0a	0.2 ± 0a	0.3 ± 0.1a	0.3 ± 0a
*p*-Cumaric acid	0.3 ± 0.1a	0.3 ± 0a	0.4 ± 0a	0.4 ± 0a
3,4-DHPEA-EDA	536.3 ± 12ac	570.7 ± 3.3a	417.8 ± 13.6b	481.7 ± 22.3c
*p*-HPEA-EDA	62.1 ± 3.9a	60.7 ± 3.9a	56 ± 1.1a	57.5 ± 2.1a
(+)-1-acetoxypinoresinol	29.8 ± 0.6a	28.2 ± 0.7a	30.1 ± 0.3a	23.6 ± 9.7a
(+)-pinoresinol	3.7 ± 0.5a	7.3 ± 5.8a	15.1 ± 0.9a	13.5 ± 0.3a
3,4-DHPEA-EA	71.2 ± 4.6a	97.6 ± 6.4b	99.6 ± 2.9b	93.8 ± 4.7b
Ligstroside aglycone	15.2 ± 0.2a	12.7 ± 0.8a	14.7 ± 3.1a	14.2 ± 0.2a
Total phenols	720.8 ± 14.2ac	780.2 ± 12a	636.7 ± 14.6b	687.7 ± 25.3bc
	Peranzana			
3,4-DHPEA ^1^	1.1 ± 0.1a	0.9 ± 0.4a	0.9 ± 0.1a	1 ± 0.2a
*p*-HPEA	3.2 ± 0.4a	2.3 ± 0.6a	2 ± 0.3a	3 ± 0.5a
Vanilic acid	0.5 ± 0.1a	0.3 ± 0.2a	0.2 ± 0.1a	0.6 ± 0.2a
*p*-Cumaric acid	0.3 ± 0a	0.2 ± 0.1a	0 ± 0b	0 ± 0b
3,4-DHPEA-EDA	231.2 ± 1.6ac	279.6 ± 6.1b	213.8 ± 13.1a	240.5 ± 7.3a
*p*-HPEA-EDA	70.4 ± 2.4a	71.8 ± 3.9a	60.2 ± 1.1b	60.4 ± 0.7b
(+)-1-acetoxypinoresinol	10.8 ± 2.5a	7.9 ± 0.1a	5.6 ± 0.4a	6.8 ± 0.4a
(+)-pinoresinol	4.6 ± 0.5a	4.3 ± 0.3a	4.5 ± 0.3a	4.2 ± 0.2a
3,4-DHPEA-EA	53.5 ± 0.5ab	63.9 ± 4.4a	50.2 ± 4b	63.5 ± 2.9ab
Ligstroside aglycone	12.1 ± 0.7a	13.9 ± 1.8a	12.4 ± 1.1a	13 ± 1.1a
Total phenols	387.6 ± 4ac	445 ± 9.8b	349.7 ± 14.3a	393.1 ± 8.5c

^1^ The data are the mean values of two independent extractions, ± standard deviation. The values in each row with different letters (a–d) are significantly different from one another (*p* < 0.05). C = control (heat exchanger at 25 °C, malaxer at 25 °C for 30 min); T1 = test 1 (heat exchanger at 18 °C, malaxer at 25 °C for 30 min); T2 = test 2 (heat exchanger at 18 °C, malaxer at 18 °C for 30 min); T3 = test 3 (heat exchanger at 18 °C, malaxer at 18 °C for 15 min + 15 min at 25 °C).

**Table 3:** Volatile composition (µg/kg) of Canino VOOs extracted at different temperatures.

	C-VOO	T1-VOO	T2-VOO	T3-VOO
Aldehydes ^1^				
Pentanal	13 ± 1a	13 ± 2a	15 ± 1a	10 ± 1a
(E)-2-Pentenal	24 ± 1a	24 ± 2a	35 ± 3b	26 ± 2ab
Hexanal	1072 ± 79a	1075 ± 78a	1124 ± 36a	961 ± 37a
(E)-2-Hexenal	23635 ± 1162a	26416 ± 1671ac	35712 ± 2257b	31580 ± 987bc
(E,E)-2,4-Hexadienal	110 ± 7a	120 ± 16a	120 ± 5a	87 ± 0a
Ʃ of aldehydes	24855 ± 1164a	27647 ± 1673ac	37006 ± 2257b	32665 ± 988bc
Alcohols				
1-Pentanol	37 ± 3a	22 ± 2bc	26 ± 2b	16 ± 0c
1-Penten-3-ol	96 ± 9a	103 ± 11bc	139 ± 8b	165 ± 18c
(E)-2-Penten-1-ol	11 ± 1a	12 ± 1a	15 ± 2a	39 ± 3b
(Z)-2-Penten-1-ol	91 ± 5a	101 ± 7ab	134 ± 12bc	153 ± 8c
1-Hexanol	252 ± 12a	180 ± 18b	129 ± 7b	184 ± 19b
(E)-2-Hexen-1-ol	652 ± 28a	669 ± 35a	367 ± 24b	517 ± 14c
(Z)-3-Esen-1-olo	99 ± 9a	102 ± 5a	125 ± 8a	182 ± 8b
Benzyl alcohol	69 ± 8a	74 ± 6a	77 ± 1a	71 ± 1a
Pheniletyl alcohol	32 ± 3a	32 ± 3a	45 ± 1b	39 ± 3ab
Ʃ of C_5_ and C_6_ alcohols	1238 ± 33a	1189 ± 41a	935 ± 30b	1256 ± 32a
Esters				
Hexyl acetate	14 ± 0a	8 ± 1b	5 ± 1b	11 ± 1a
(Z)-3-Hexenyl acetate	9 ± 1a	6 ± 1ab	7 ± 0ab	6 ± 1b
Ʃ of esters	23 ± 1a	14 ± 1bc	11 ± 1b	17 ± 1c
Ketones				
3-Pentanone + 2-Pentanone	32 ± 2a	32 ± 2a	31 ± 3a	31 ± 1a
1-Penten-3-one	195 ± 14a	181 ± 8a	295 ± 15b	230 ± 14a
6-Methyl-5-hepten-2-one	11 ± 0a	10 ± 1a	8 ± 1a	5 ± 0b
Ʃ of ketones	238 ± 14a	223 ± 8a	334 ± 15b	266 ± 14a

^1^ The data are the mean values of two independent extractions, ± standard deviation. The values in each row with different letters (a–d) are significantly different from one another (*p* < 0.05). C = control (heat exchanger at 25 °C, malaxer at 25 °C for 30 min); T1 = test 1 (heat exchanger at 18 °C, malaxer at 25 °C for 30 min); T2 = test 2 (heat exchanger at 18 °C, malaxer at 18 °C for 30 min); T3 = test 3 (heat exchanger at 18 °C, malaxer at 18 °C for 15 min + 15 min at 25 °C).

**Table 4:** Volatile composition (µg/kg) of Moraiolo VOOs extracted at different temperature.

	C-VOO	T1-VOO	T2-VOO	T3-VOO
Aldehydes ^1^				
Pentanal	18 ± 2a	18 ± 1a	24 ± 3a	22 ± 0a
(E)-2-Pentenal	52 ± 5a	44 ± 4a	45 ± 12a	49 ± 2a
Hexanal	830 ± 1a	865 ± 53a	903 ± 94a	657 ± 68a
(E)-2-Hexenal	22141 ± 800a	22432 ± 719a	24060 ± 1344a	22091 ± 1320a
(E,E)-2,4-Hexadienal	86 ± 5ab	75 ± 6a	99 ± 4b	96 ± 7ab
Ʃ of aldehydes	23128 ± 800a	23435 ± 721a	25131 ± 1348a	22915 ± 1321a
Alcohols				
1-Pentanol	23 ± 3a	20 ± 4a	22 ± 1a	19 ± 2a
1-Penten-3-ol	294 ± 21a	262 ± 11ab	216 ± 7b	274 ± 7a
(E)-2-Penten-1-ol	30 ± 2a	24 ± 5a	19 ± 2a	27 ± 1a
(Z)-2-Penten-1-ol	307 ± 21a	280 ± 14ab	223 ± 15b	290 ± 4a
1-Hexanol	450 ± 7a	467 ± 18a	549 ± 52a	450 ± 35a
(E)-2-Hexen-1-ol	679 ± 8a	551 ± 21bc	515 ± 33b	665 ± 41ac
(Z)-3-Esen-1-olo	449 ± 42a	426 ± 41a	515 ± 12a	460 ± 39a
Benzyl alcohol	67 ± 2a	75 ± 8a	85 ± 8a	77 ± 3a
Pheniletyl alcohol	78 ± 1a	93 ± 7a	87 ± 5a	75 ± 4a
Ʃ of C_5_ and C_6_ alcohols	2231 ± 53a	2031 ± 52a	2059 ± 65a	2184 ± 67a
Esters				
Hexyl acetate	5 ± 0a	5 ± 0a	5 ± 1a	4 ± 0a
(Z)-3-Hexenyl acetate	5 ± 0a	6 ± 1a	17 ± 2b	8 ± 1a
Ʃ of esters	10 ± 1a	12 ± 1a	22 ± 2b	13 ± 1a
Ketones				
3-Pentanone + 2-Pentanone	121 ± 4a	103 ± 13a	114 ± 8a	98 ± 10a
1-Penten-3-one	513 ± 20a	410 ± 7b	324 ± 25c	453 ± 16ab
6-Methyl-5-hepten-2-one	6 ± 0a	7 ± 1a	8 ± 1a	7 ± 1a
Ʃ of ketones	640 ± 20a	520 ± 14bc	445 ± 27b	558 ± 19ac

^1^ The data are the mean values of two independent extractions, ± standard deviation. The values in each row with different letters (a–d) are significantly different from one another (*p* < 0.05). C = control (heat exchanger at 25 °C, malaxer at 25 °C for 30 min); T1 = test 1 (heat exchanger at 18 °C, malaxer at 25 °C for 30 min); T2 = test 2 (heat exchanger at 18 °C, malaxer at 18 °C for 30 min); T3 = test 3 (heat exchanger at 18 °C, malaxer at 18 °C for 15 min + 15 min at 25 °C).

**Table 5:** Volatile composition (µg/kg) of Peranzana VOOs extracted at different temperatures.

	C-VOO	T1-VOO	T2-VOO	T3-VOO
Aldehydes ^1^				
Pentanal	17 ± 0a	10 ± 1b	15 ± 1a	12 ± 2a
(E)-2-Pentenal	68 ± 3a	42 ± 18a	55 ± 26a	38 ± 1a
Hexanal	925 ± 59a	1321 ± 12a	1388 ± 481a	1150 ± 144a
(E)-2-Hexenal	17306 ± 1308a	15181 ± 1771a	18043 ± 476a	17045 ± 935a
(E,E)-2,4-Hexadienal	133 ± 13a	139 ± 30a	143 ± 61a	123 ± 33a
Ʃ of aldehydes	18450 ± 1309a	16693 ± 1772a	19644 ± 680a	18368 ± 947a
*Alcohols*				
1-Pentanol	18 ± 3a	20 ± 1a	19 ± 0a	17 ± 2a
1-Penten-3-ol	340 ± 11a	325 ± 20a	283 ± 168a	166 ± 3a
(E)-2-Penten-1-ol	28 ± 0a	16 ± 9a	21 ± 12a	13 ± 0a
(Z)-2-Penten-1-ol	360 ± 6a	252 ± 51ab	178 ± 24b	150 ± 15b
1-Hexanol	147 ± 7a	103 ± 8a	96 ± 37a	64 ± 8a
(E)-2-Hexen-1-ol	143 ± 22a	107 ± 7ab	86 ± 8b	74 ± 9b
(Z)-3-Esen-1-olo	282 ± 9a	161 ± 21a	177 ± 112a	91 ± 9a
Benzyl alcohol	71 ± 3a	70 ± 7a	88 ± 31a	63 ± 4a
Pheniletyl alcohol	61 ± 8a	38 ± 3a	45 ± 23a	27 ± 2a
Ʃ of C_5_ and C_6_ alcohols	1318 ± 27a	985 ± 60ab	860 ± 207b	575 ± 22b
Esters				
Hexyl acetate	8 ± 4a	52 ± 19ab	28 ± 16ab	65 ± 12b
(Z)-3-Hexenyl acetate	124 ± 6a	148 ± 21a	168 ± 9a	159 ± 10a
Ʃ of esters	132 ± 7a	201 ± 28ab	196 ± 19ab	224 ± 16b
Ketones				
3-Pentanone + 2-Pentanone	26 ± 1a	13 ± 7a	25 ± 4a	11 ± 2a
1-Penten-3-one	662 ± 29a	367 ± 169ab	270 ± 25b	680 ± 40a
6-Methyl-5-hepten-2-one	8 ± 0a	8 ± 1a	8 ± 0a	9 ± 0a
Ʃ of ketones	696 ± 29a	389 ± 169ab	303 ± 25b	699 ± 40a

^1^ The data are the mean values of two independent extractions, ± standard deviation. The values in each row with different letters (a–d) are significantly different from one another (*p* < 0.05). C = control (heat exchanger at 25 °C, malaxer at 25 °C for 30 min); T1 = test 1 (heat exchanger at 18 °C, malaxer at 25 °C for 30 min); T2 = test 2 (heat exchanger at 18 °C, malaxer at 18 °C for 30 min); T3 = test 3 (heat exchanger at 18 °C, malaxer at 18 °C for 15 min + 15 min at 25 °C).
